# Plant SILAC: Stable-Isotope Labelling with Amino Acids of *Arabidopsis* Seedlings for Quantitative Proteomics

**DOI:** 10.1371/journal.pone.0072207

**Published:** 2013-08-20

**Authors:** Dominika Lewandowska, Sara ten Have, Kelly Hodge, Vinciane Tillemans, Angus I. Lamond, John W. S. Brown

**Affiliations:** 1 Cell and Molecular Sciences, The James Hutton Institute, Dundee, United Kingdom; 2 Division of Plant Sciences, College of Life Sciences, University of Dundee at the James Hutton Institute, Dundee, United Kingdom; 3 Centre for Gene Regulation and Expression, College of Life Sciences, University of Dundee, Dundee, United Kingdom; University of Hyderabad, India

## Abstract

Stable Isotope Labelling by Amino acids in Cell culture (SILAC) is a powerful technique for comparative quantitative proteomics, which has recently been applied to a number of different eukaryotic organisms. Inefficient incorporation of labelled amino acids in cell cultures of *Arabidopsis thaliana* has led to very limited use of SILAC in plant systems. We present a method allowing, for the first time, efficient labelling with stable isotope-containing arginine and lysine of whole Arabidopsis seedlings. To illustrate the utility of this method, we have combined the high labelling efficiency (>95%) with quantitative proteomics analyses of seedlings exposed to increased salt concentration. In plants treated for 7 days with 80 mM NaCl, a relatively mild salt stress, 215 proteins were identified whose expression levels changed significantly compared to untreated seedling controls. The 92 up-regulated proteins included proteins involved in abiotic stress responses and photosynthesis, while the 123 down-regulated proteins were enriched in proteins involved in reduction of oxidative stress and other stress responses, respectively. Efficient labelling of whole Arabidopsis seedlings by this modified SILAC method opens new opportunities to exploit the genetic resources of Arabidopsis and analyse the impact of mutations on quantitative protein dynamics *in vivo*.

## Introduction

Stable Isotope Labelling by Amino acids in Cell culture (SILAC) is a mass spectrometry-based quantitative proteomic technology, originally developed to measure changes in relative protein levels in mammalian tissue culture cells grown under different experimental conditions. Cells are usually metabolically labelled by incorporation of the stable isotope-containing amino acids arginine and lysine, which are supplied in the culture medium. Thus, following protein isolation and trypsin digestion, all peptides terminate in a single labelled amino acid [1]. SILAC allows the routine identification and accurate large-scale quantification of hundreds to thousands of proteins usually by identification of several unique peptides [1]–[3]. The expected mass differences of ‘heavy’ and ‘light’ peptides are known before their identification and quantitation of proteins by MaxQuant is relatively straightforward. It has been widely used in animal systems, primarily in cell cultures, but more recently has also been applied to labelling multicellular model organisms, such as mouse [4] and *Caenorhabiditis elegans* [5], [6].

Proteomics studies in plants have used different comparative proteomic technologies (metabolic labelling, chemical post-processing labelling or label-free) to identify dynamic changes in proteins [7], [8]. All have advantages and disadvantages [9]. In terms of metabolic labelling in plants, ^15^N labelling has become the method of choice. ^15^N labelling allows two-way treatments to be compared. The stable isotope is introduced into the growth media as an inorganic ^15^N-containing salt as the sole nitrogen source for the plant and has been successfully used to label plant cell cultures [10]–[13]. Moreover, efficient ^15^N labelling *in planta* can be obtained in hydroponically grown plants [14]–[16], plants grown on a solid media [17] or even in the soil (SILIP technique) [18]. The main disadvantages of ^15^N labelling are suboptimal labelling, so the ^14^N/^15^N ratio must be taken into account during quantification. Peptide mass differences are also unknown prior to identification requiring MS/MS peptide sequencing for identification, and sensitivity may make identification, and therefore quantification of low abundance peptides, difficult.

SILAC has generally been regarded as unsuitable for plant systems, mainly due to poor metabolic labelling efficiency, which affects the accuracy of quantification of peptide ratios. To date, there are only two reports of SILAC labelling in plant systems, both using Arabidopsis cell cultures, which were labelled with ca. 80% and 83–91% efficiency, respectively, making quantitation complex [19], [20]. One of the main disadvantages of SILAC is that suboptimal labelling efficiencies of autotrophs can affect sensitivity and accuracy of quantitation of proteins. To overcome the problem of suboptimal labelling, both control and treated cell cultures were labelled with different isotopes, which allowed relative quantitation of labelled peptides and improved accuracy of quantitation [20]. So far, the only organism from the plant kingdom that has been successfully and efficiently SILAC labelled is an auxotrophic mutant of *Chlamydomonas reinhardtii* [21]. Here, we present a straightforward method adapting the SILAC procedure [3] for *Arabidopsis thaliana* that allows the efficient incorporation of stable isotope-labelled amino acids into the proteomes of whole Arabidopsis seedlings. This method routinely provides >95% incorporation of stable isotope-labelled amino acids in three-week old seedlings.

## Materials and Methods

### Materials

Complete protease inhibitor cocktail tablets were from Roche. A Bicinchoninic Acid Assay (BCA) Kit was from Pierce. InstantBlue staining kit was from Expedeon. Trypsin was from Promega. C18 cleaning columns were from Applied Biosystems and the Pepmap C18 columns were from Dionex. All other materials were obtained from Sigma.

### *Arabidopsis Thaliana* Seedling Growth Conditions

**Figure 1:**
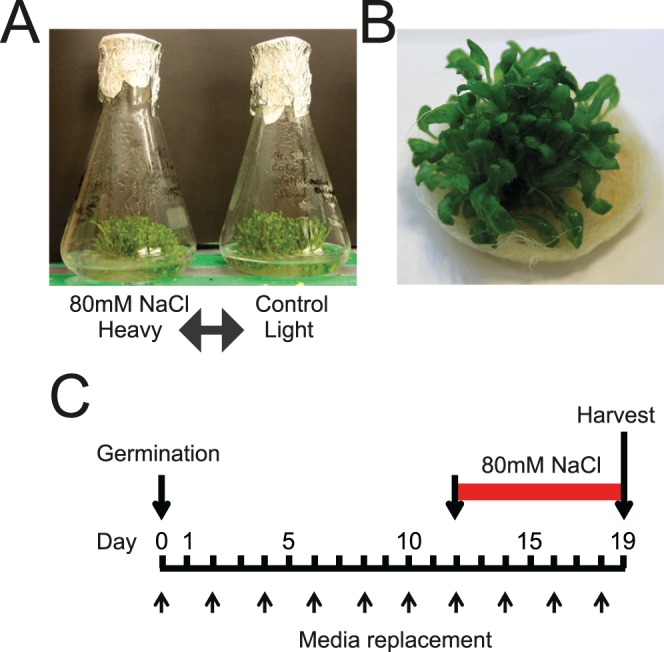
Experimental system for SILAC labelling of Arabidopsis seedlings. Seedlings were grown from seeds germinated in liquid medium with reciprocal labelling of salt-treated and control plants (A); seedlings prior to harvest formed an island of material which floated on the medium with roots immersed (B); experimental plan showing days after germination, media changes, salt treatment duration and time of harvest (C).

*Arabidopsis thaliana* ecotype Col-0 was used for all experiments. Around 15 mg of Arabidopsis seeds were sterilized with 0.6% sodium hypochlorite for 5 minutes, followed by multiple washes with distilled sterile water. Seeds were germinated in liquid medium with shaking. After germination (around 3–4 days) the seedlings remain on the surface of the medium and form islands or rafts of plantlets where the roots are immersed but shoots are above the surface of the medium (Fig. 1A and B). Seedlings were grown in 25 ml liquid culture with Gamborg’s medium, consisting of 3.2 g/l Gamborg’s B5 salts+minimal organics, 1 ml/l 1000×Gamborg’s vitamins, 0.5 g/l MES, 3% sucrose, pH 5.9 and supplemented with 160 µg/ml L-Lysine and 160 µg/ml L-Arginine in a 250 ml flask (Fig. 1A). Seedlings were grown at 22°C in a 16 h light/8 h dark cycle and with vigorous shaking (around 120 rpm) for 19 days (from seed germination). Medium was exchanged frequently (every two days after the seeds have germinated) giving a total number of 8–9 media changes through the time of the culture growth. For the salt stress treatment, Gamborg’s medium containing 80 mM NaCl was used from the 12th day of culturing (8 days after germination) for 7 days (Fig. 1C).

### SILAC Labelling

To achieve efficient labelling of Arabidopsis seedlings, SILAC medium was used, where L-arginine was substituted with L-arginine (U-^13^C_6_, 99%) (Arg6) (160 mg/l) and L-lysine was substituted with L-lysine:2HCL (^4,4,5,5^-D_4_, 96–98%) (Lys4) (160 mg/l) (Cambridge Isotope Laboratories) in the growth medium. Seeds were germinated in isotope-containing SILAC medium and seedlings were grown in the same conditions as described above with the SILAC medium being changed every 2 days. Six biological repeats of reciprocally labelled salt-treated and control plants were performed. Each repeat consisted of a pair of label swap treatments (e.g. control with label, salt treatment without label; or control without label, salt treatment with label).

### Protein Extraction

Arabidopsis plants were collected from culture flasks, dried from the liquid medium using paper towels, shoot material harvested and flash frozen in the liquid nitrogen. For total protein extract preparation, 1 g of the frozen heavy-isotope labeled (‘heavy’) culture and 1 g of frozen light-isotope labeled (‘light’) culture was used. For lysis, the frozen tissue was ground in a mortar and pestle and extracted with 1.5 ml of extraction buffer (50 mM Tris HCl pH 7.6, 0.33M sucrose, 1 mM MgCl_2_, 1 mM DTT, 1% (w/v) C7BzO, protease inhibitor cocktail) for 45 minutes on ice. The lysates were then centrifuged for 10 min at 4000×g at 4°C. Supernatants were collected in fresh tubes and centrifuged again for 10 min at 18000×g at 4°C. A Bicinchoninic Acid Assay (BCA) was performed on the supernatants to determine protein concentration; equal proportions of protein from ‘heavy’ and ‘light’ cultures were combined.

### Sample Preparation

Size fractionation of the combined proteins was achieved by SDS-PAGE analysis on 4–12% (w/v) Bis-Tris NuPage gels using 4-Morpholinepropanesulfonic acid (MOPS) running buffer (Invitrogen) according to manufacturer’s instructions, in the LDS NuPage sample buffer. A maximum of 20 µg of protein was loaded per lane. InstantBlue staining was performed according to manufacturer’s instructions (Expedeon). Each lane from the gel was cut into 10 fractions and gel pieces de-stained and proteins were reduced with 10 mM DTT and alkylated with 55 mM iodoacetamide. The gel slices were then treated with trypsin (due to undigested material present after the standard double digestion with trypsin, a triple digest protocol was adopted, with one digest done overnight at 37°C, followed by fresh trypsin aliquot addition, 4 hours shaking at 37°C, and repeated). The resulting peptides were cleaned over a C18 (POROS R2, Applied Biosystems) column. The column was first activated with 50% acetonitrile, 0.1% Trifluoro acetic acid (TFA) and then washed with 0.1% TFA. Sample was loaded onto the column and washed with 0.1% TFA. Bound peptides were eluted from the column using 50% acetonitrile, 0.1% TFA. Samples were dried down to approximately 10 µl using vacuum centrifugation.

### LC-MS/MS and MaxQuant Analysis

A Dionex Ultimate 3000 nanoHPLC system was used with 2 µg of peptides injected onto an Acclaim PepMap C18 nano-trap column (Dionex). After washing with 2% (vol/vol) acetonitrile 0.1% (vol/vol) formic acid peptides were resolved on a 150 mm × 75 µm Acclaim PepMap C18 reverse phase analytical column over a 200 min organic gradient with a flow rate of 300 nl min−1. The chromatography performed for these samples was as follows. The gradient commenced with 4 minutes of 95% buffer A (0.1% formic acid)/5% buffer B (80% acetonitrile, 0.08% formic acid), followed by a linear gradient to 40% buffer B over 128 minutes, then an increase to 98% buffer B for 20 minutes duration, and completed with a return to 5% buffer B at minute 152 for 30 minutes. Ions accepted for MS/MS were 2+ and greater. Dynamic exclusion was set to 45 seconds, and the inclusion mass width for precursor ions was 10 ppm. The allowed number of missed trypsin cleavages was set to 2. Peptides were ionized by nano-electrospray ionization at 1.2 kV using a fused silica emitter with an internal diameter of 5 µm (New Objective). Tandem mass spectrometry analysis was carried out on a LTQ-Velos Orbitrap mass spectrometer (Thermo Scientific) using data-dependent acquisition, measuring and sequencing the top 15 ions.

The resulting raw files were processed, quantified and searched using MaxQuant version 1.3.0.51 and the Andromeda peptide search engine [22], [23], searching against the Uniprot *Arabidopsis thaliana* database (updated September 2012). The variable modifications were set as oxidation of methionine; acetylation of the protein N-terminus; deamidation of asparagine and glutamine, glutamine conversion to pyroglutamate; as well as the heavy proline products to determine if loss of heavy label was due to arginine conversion. Fixed modifications were set to carbamidomethylation of cysteines only. The MS tolerance was set to 7 ppm with the MS/MS tolerance set to 0.5 Da. The peptide and protein False Discovery Rate (FDR) were both set to 1% [23], and the proteins used for quantitation and further analysis had 2 or more peptides assigned to them. Significance of fold changes was calculated using a one sample Benjamini-Hochberg t-test analysis with a 0.05 threshold value.

### Quality Control of Labelling in Different Experiments

The biological repeats were first analysed for labelling efficiency as the reciprocal label swap needed to verify the null effect of the SILAC labelling on the plants. This was done by graphing the log_2_ ratios of the entire population of proteins and determining their normalised distribution centred over 0 (in this case slightly off 0 as the protein quantification of chlorophyll containing protein solutions is problematic, thus we saw a consistent mixing error). The normalisation of the mixing error was done using MaxQuant software [23], although this did not alter the labelling efficiency estimations, and these were consistently comparable to mammalian cell culture. Correlations of datasets were performed and any non-correlating data was identified resulting in one biological repetition being excluded from further analysis.

### Protein Function and Gene Ontology Analysis

To examine the distribution of up- and down-regulated proteins, gene identifiers were entered into the Plant Protein database (PPDB - http://ppdb.tc.cornell.edu/) and grouped by function using the MapMan system. To assess the degree to which protein classifications were over-represented compared to Arabidopsis proteins as a whole, the up- and down-regulated genes were submitted to enrichment analysis using the DAVID Functional Annotation tool (http://david.abcc.ncifcrf.gov/home.jsp). Gene Ontology (GO) terms relating to biological process, cellular compartment, and molecular function were identified with *p*<0.05.

## Results

### Efficient Incorporation of Stable Isotope Labelled Amino Acids

The major problem with the application of SILAC to plants has been limited incorporation of stable-isotope-containing amino acids, thought to be due to plants being autotrophic. Indeed, successful SILAC in *Chlamydomonas* was achieved by generating an arginine auxotrophic mutant [21]. To establish SILAC in plants, we therefore examined whether mutants in the arginine and lysine synthesis pathways would increase the efficiency of labelling with stable isoptope-containing Arg and Lys. The L-arginine and L-lysine biosynthesis pathways in Arabidopsis are well understood [24], [25]. We investigated four (SALK_042885 for At3g53580, SALK_095812 for At3g53580, SALK_044782 for At4g33680, CATMA4a35440 for At4g33680) and six (SALK_047105 for At1g29900, SALK_038130 for At4g24830, SALK_070991 for At4g37670, SALK_070983 for At4g37670, SALK_138081 for At2g19940, SALK_085035 for At2g22910) knock-out mutants (T-DNA insertions) in genes in the lysine and arginine biosynthetic pathways, respectively. Some mutant lines showed poor growth phenotypes under normal conditions and some of the arginine pathway mutants had reduced levels of arginine in preliminary HPLC analyses (results not shown). However, in SILAC experiments, none of the mutants showed significantly higher levels of ‘heavy’ arginine and lysine incorporation when compared to wild-type Arabidopsis seedlings (results not shown) and subsequent experiments were therefore performed with wild-type plants. We also attempted to label Arabidopsis cell cultures but obtained lower incorporation consistent with previous studies [19], [20].

**Figure 2:**
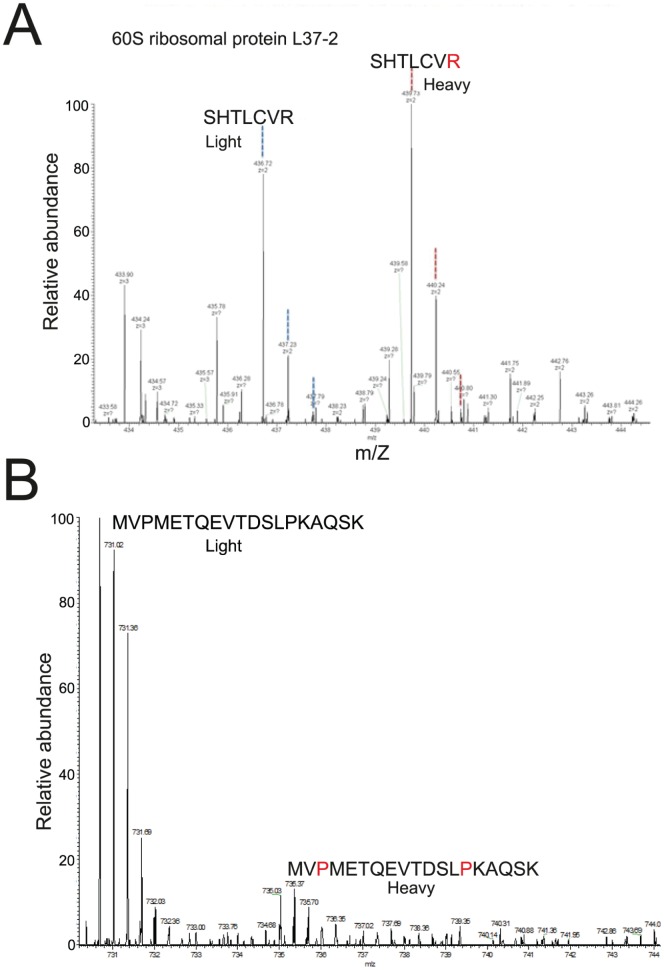
Efficient SILAC labelling of Arabidopsis seedlings and assessment of arginine-proline conversion. Mass spectra of unlabelled (“light”) and labelled (“heavy”) peptides of L37-2 ribosomal protein showing efficient labelling (SILAC ratio = 1.0023) (A). Mass spectra of “light” and “heavy” (heavy proline) peptides of an RNA helicase protein (B).

Seedlings were germinated on basal medium with minimal organics containing ^13^C_6_-arginine and ^4^H_2_-lysine and grown for up to 23 days, with the medium containing isotope-labelled amino acids being changed every two days throughout the growth period (Figure 1A–C). Shoot material from the heavy-isotope labelled (‘heavy’) and unlabelled (‘light’) seedlings was harvested and frozen in liquid nitrogen. Total protein was extracted and equal amounts of protein from ‘heavy’ and ‘light’ shoot material were combined and size fractionated by SDS-PAGE and treated with trypsin. Peptides were subjected to tandem mass spectrometry analysis on a LTQ-Velos Orbitrap mass spectrometer (Thermo Scientific); peptide data were quantified and proteins identified against the Uniprot Arabidopsis thaliana database (updated September 2012) using MaxQuant. The efficiency of label incorporation in seedlings (measured by MS) was >95% as seen for representative peptides (Figure 2A). This level of label incorporation is sufficient for quantitation of peptides by MaxQuant and is comparable with typical labelling efficiencies in human cell cultures. The reasons for the high labelling efficiency obtained with whole seedlings, as compared to Arabidopsis cell cultures, may reflect 1) the frequent changes of culture medium, such that high levels of labelled amino acids are maintained and 2) that seeds were germinated in label-containing medium, such that from the start of germination, rapidly dividing and developing seedling tissue is constantly exposed to labelled amino acids (perhaps reducing the requirement for autotrophic amino acid production).

### Low Relative Levels of Arginine-proline Conversion

A potential problem with SILAC is the *in vivo* metabolic conversion of arginine to proline, which can affect accurate quantitation of peptides. The presence of heavy proline-containing peptides reduces the signal of heavy arginine labelled peptides, leading to an underestimate of the heavy/light peptide ratio [3]. In mammalian cells, arginine conversion can occur in 10–15% of the proline pool [26] and around 30–40% of proline-containing peptides can contain heavy proline [27]. To assess the degree of arginine-proline conversion we searched for peptides that contained heavy proline in 4 out of 5 of the biological replicates. We identified only 32 different heavy proline-containing peptides from the many thousands of sequenced peptides. For example, one such peptide (MVPMETQEVTDSLPKAQSK) was from an ATP-dependent RNA helicase-like protein (At2g28240). It was not labelled with heavy lysine and all occurrences contained heavy proline, but never with a high ratio (the averaged ratio over 5 biological reps was 0.14) (Figure 2B). Overall, arginine-proline conversion was approximately 6% (comparing the total number of heavy proline-containing peptides to the total number of proline-containing peptides) and peptides containing heavy proline were all present in relatively low ratios.

### Identification of Up- and Down-regulated Proteins in Response Salt Treatment

To demonstrate the utility of SILAC labelling of whole seedlings to address dynamic changes in protein composition, we examined the response in shoots of seedlings exposed to modest salt stress [28]. Seedlings were grown in medium containing 80 mM NaCl for 7 days (from day 12 to 19 of culture/8–15 days post-germination) (Figure 1C). Label swap experiments for control and salt-treated seedlings were performed, giving a total of six biological repetitions. During the routine correlation analysis of all of the datasets (label swap experiments should give positive and negative correlations) one repeat did not give good correlation and thus five biological repeats were analysed. No phenotypic differences were observed between labelled and non-labelled untreated seedlings. Chlorosis of young leaves was previously observed in Arabidopsis plants watered with 50 or 150 mM NaCl [8] but we did not observe phenotypic differences between salt-treated (80 mM for 7 days) and untreated seedlings at the time of harvest, although some yellowing of leaves was seen at around 23 days of culture.

**Figure 3:**
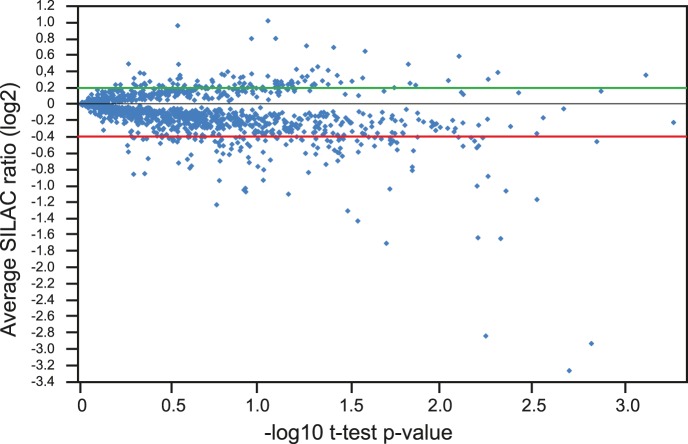
Changes in the proteome of salt-treated Arabidopsis seedlings. Quantification of protein changes in response to 80 mM NaCl - the relative fold increase or decrease of all of the proteins analysed is indicated on the *y* axis expressed as the log_2_ of the average SILAC ratio across the five bioreps plotted against –log_10_ of the t-test *p*-value. For further analysis, up- and down-regulated proteins were taken as those outside the cut-off of 0.2 to −0.4.

**Figure 4:**
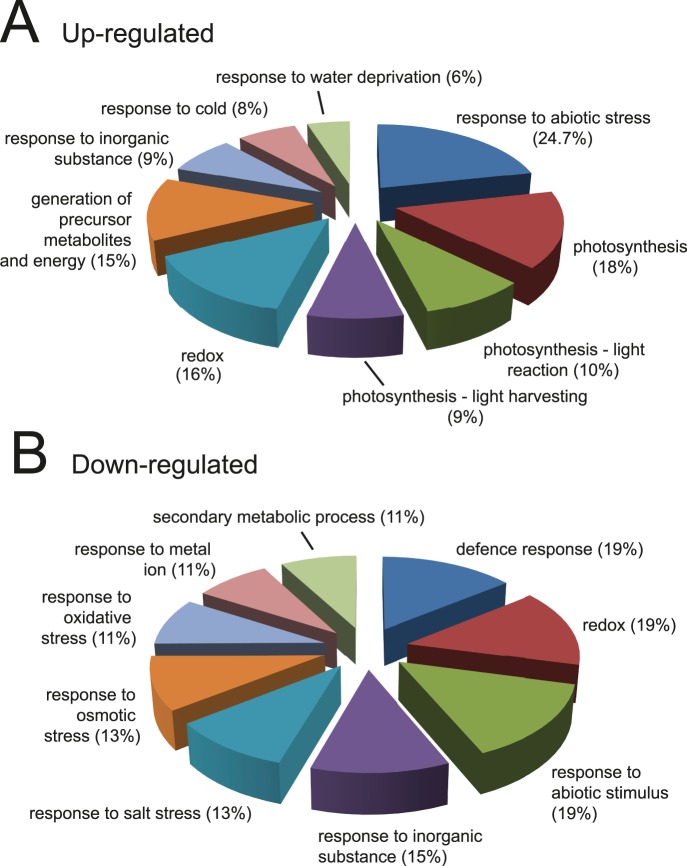
Distribution of up- and down-regulated proteins by biological process. Gene ontology (GO) term enrichment distribution of up-regulated proteins (A) and down-regulated proteins (B) using DAVID. Frequency distribution (%) of proteins in significantly enriched (*p*<0.05) GO:Biological Process function terms are given (see also Table S4 and Table S5).

We identified a total of 2,858 proteins with a minimum of two peptides (14,347 unique peptides/22,991 peptides in total). Following quantification using MaxQuant [23], 92 and 123 proteins were consistently either up- or down-regulated by the saline treatment, respectively, in at least four of the five biological replicates (Figure 3; Tables S1–S3). The functions of the identified proteins were analysed using the DAVID Functional Enrichment Chart Tool (http://david.abcc.ncifcrf.gov/content.jsp?file=functional_annotation.html). Enrichment of gene ontology (GO):Biological Process terms showed over-representation of proteins involved in photosynthesis amongst up-regulated proteins (Figure 4A) whereas in the down-regulated protein set, terms associated with various stress responses and cell wall enzymes were over-represented (*p*<0.05) (Figure 4B). A similar analysis using the Plant Protein database (PPDB - http://ppdb.tc.cornell.edu/) showed up-regulation of chloroplast and photosynthetic machinery, including RuBisCo, components of photosystems I and II and enzymes involved in tetrapyrrole synthesis for chlorophyll (Figure S1). Consistent with increased photosynthetic capacity, enzymes involved in deactivating reactive oxygen species (e.g. glutathione-S-transferase, peroxidases and thioredoxins) were down-regulated (Figure S1) [28].

## Discussion

We have developed a method for the application of SILAC to Arabidopsis seedlings, which allows, for the first time, SILAC proteomics to be used in whole plants. SILAC labelling of Arabidopsis cell cultures was relatively inefficient [19], [20] and therefore we expected that efficient labelling of seedlings would require the use of auxotrophic mutants in the lysine and arginine pathways. Although, we identified such mutants, we did not find any significant advantage in their use over wild type seedlings in our stable isotope labelling regime, with >95% incorporation. This level of incorporation is sufficient for the application of SILAC to studies in Arabidopsis. It will permit direct comparison of dynamic changes in the proteomes of plants grown under, for example, different abiotic and biotic stress conditions. In addition, as auxotrophic mutants are not required to increase label incorporation, this method can be applied directly to the examination of Arabidopsis mutants.

We have demonstrated the SILAC labelling of whole seedlings by analysing the response to moderate salt stress. Arabidopsis, a glycophyte, is sensitive to salt stress, which inhibits growth and ultimately leads to senescence and death. Responses to increased salinity occur in two distinct phases: a rapid onset osmotic phase, which induces stomatal closing, inhibits growth of young leaves and is independent of Na^+^ accumulation in the shoot, and a slower ionic phase as Na^+^ gradually accumulates in older leaves causing premature senescence [28]. The two phases have distinct effects and responses. In general, growth inhibition due to salinity is associated with a decrease in carbon assimilation/photosynthesis, carbohydrate metabolism and cell wall production and increased expression of genes involved in scavenging reactive oxygen species (ROS), nucleotide and fatty acid metabolism, ion homeostasis, osmolyte biosynthesis/accumulation and signal transduction [28], [29].

Here, we identified 92 and 123 proteins, which were significantly up- or down-regulated in shoot material by the salt treatment compared to plants without salt treatment. In shoots treated for 8 days with 80 mM NaCl, chloroplast structural proteins, photosynthetic and light-responsive proteins, and some abiotic stress response proteins were enriched among up-regulated proteins while ROS-inactivating proteins and other biotic and abiotic stress response proteins including salt and osmotic stress were down-regulated (Figure S1; Table S4 and Table S5). On a general level, these changes appear to contrast those from other proteomic studies of salt responses (see [29]). However, it is also clear that proteins identified in proteomic analyses of responses to saline conditions are highly variable and most likely reflect variation in experimental conditions, such as salt concentration, duration of treatment, method of application, cell culture versus whole seedlings, age of seedlings/plants, or type of material (e.g. shoots and/or roots) analysed. For example, variation in proteomic responses to salt stress is illustrated by comparing the regulated proteins found here by SILAC to those of previous salt stress responses in Arabidopsis determined mainly by 2-dimensional gel electrophoresis/MS [8], [30], [31] (Figure S2; Table S6 and S7). There was only small overlap in the identity of both up- and down-regulated proteins with the majority being uniquely identified in the different experiments. In addition, the behaviour of some proteins in terms of whether they increased and decreased in abundance, varied among the experimental systems and even within experiments where some proteins were found to increase or decrease in levels at different salt concentrations or different lengths of exposure (Table S7) [8], [31].

Nevertheless, some up- and down-regulated proteins either showed similar behaviour to that reported in other salt treatment studies or were also observed to either increase or decrease in other stress conditions. For example, AKR4C9 (At2g37770), a chloroplast-localised aldo-keto reductase, which acts as a detoxifying enzyme by reducing a range of toxic aldehydes and ketones produced during stress, was up-regulated here and its expression is highly stimulated by many types of stress, including water deficit, salinity, cold and oxidative stress [32]. Within the group of up-regulated proteins we observed proteins involved in amino acid metabolism, including glycine dehydrogenase (At2g26080), methionine synthase (At3g03780), S-adenosyl-L-homocysteine hydrolase (At3g23810) and glycine cleavage T-protein (At1g11860), which have been also shown to be induced upon salt stress in *Chlamydomonas* [33]. One of these proteins, cysteine lyase, JR2 (At4g23600), was also identified as a salt responsive gene in *Arabidopsis thaliana* seedlings subjected to 160 mm NaCl for 4 hours, as determined by RNA-blot analysis [34]. Among the proteins whose levels decreased significantly in the salt treatment, we identified gamma-TIP1 (At2g36830), a tonoplast water channel protein involved in water transport and metabolite routing between the vacuole and cytoplasm. Transcript and protein levels of Gamma-TIP1 were also decreased significantly in response to 100 mM NaCl [35]. Finally, two proteins that increased in salt treatment: pathogenesis-related protein 5 (At1g75040) involved in defence and biotic responses and the large subunit of RuBisCo (AtCg00490), and one which decreased in salt treatment, copper chaperone (At3g56240), showed similar behaviour in the response of plants to 1 uM and 10 uM cadmium for 7 days [36]. Other proteins showed opposite effects in different stress conditions: for example, glutathione-S-transferase 7 (GST7) and osmotin-34 (a defence response protein) increased in leaves infected with *Alternaria brassicicola* but decreased in salt treatment, while chloroplast ribosome recycling factor, which dissociates the post-termination complex after translation to recycle ribosomes, increased in salt treatment, but decreased upon infection [37]. Overall, the profiles of protein expression changes identified here in response to a 7 day treatment with 80 mM NaCl might suggest that at the time of harvest, leaves are showing a degree of recovery from the initial osmotic stress prior to accumulation of Na+ to toxic levels.

The new SILAC method of labelling whole Arabidopsis seedlings has allowed the identification of >200 salt-regulated proteins. Clearly, variation exists in proteomic experiments and a systematic analysis of multiple time-points will be required to resolve the dynamic protein changes during the two phases of response to salt stress. Finally, the successful SILAC labelling of Arabidopsis seedlings demonstrated here opens opportunities for extending the method to other plant species including crop plants.

## Supporting Information

Figure S1 **Functional distribution of up- and down-regulated proteins.** Up-regulated proteins (A) and down-regulated (B) proteins were classified by function using the MapMan system at the Plant Protein database (PPDB - http://ppdb.tc.cornell.edu/) and the frequency distribution presented.
(EPS)

Figure S2 **Comparison of regulated proteins in different proteomic analyses of salt stress in Arabidopsis.** Gene lists of proteins reported as up- or down-regulated in response to salt [8], [30], [31] are compared with the SILAC results obtained here in a 4-way Venn diagram ([38] - http://bioinfogp.cnb.csic.es/tools/venny/index.html) (See also Table S6 and S7). Proteins identified in these studies were converted to At gene identifier numbers where required using BLAST to allow the comparison.
(EPS)

Table S1 **MaxQuant protein output for analysis of SILAC labelling of Arabidopsis seedlings – proteins identified in 4 out of 5 biological repetitions.** The slices correspond to the following approximate molecular weights; >250 kDa, 120–250 kDa, 95–120 kDa, 70–95 kDa, 55–70 kDa, 40–55 kDa, 36–40 kDa, 25–36 kDa, 17–25 kDa and <17 kDa (slices 1–10, 11–20, 21–30, 31–40, 41–50).
(XLSX)

Table S2 **Proteins up-regulated in salt treatment identified by SILAC in Arabidopsis seedlings (92 proteins).**
(XLSX)

Table S3 **Proteins down-regulated in salt treatment identified by SILAC in Arabidopsis seedlings (123 proteins).**
(XLSX)

Table S4 **Functional enrichment of proteins up-regulated in salt treatment using DAVID (92 proteins).**
(XLSX)

Table S5 **Functional enrichment of proteins down-regulated in salt treatment using DAVID (123 proteins).**
(XLSX)

Table S6 **Gene lists of Arabidopsis proteins which are up- and down-regulated in salt treatments from different proteomic studies.**
(XLSX)

Table S7 **Regulation of proteins common to different salt treatment proteomics studies.**
(XLSX)
